# Adolescent help-seeking: an exploration of associations with perceived cause of emotional distress

**DOI:** 10.3389/fpubh.2023.1183092

**Published:** 2023-10-02

**Authors:** Alisha O'Neill, Emily Stapley, Ishba Rehman, Neil Humphrey

**Affiliations:** ^1^Manchester Institute of Education, The University of Manchester, Manchester, United Kingdom; ^2^Evidence Based Practice Unit (EBPU), Anna Freud National Centre for Children and Families and University College London (UCL), London, United Kingdom

**Keywords:** help-seeking, adolescence, emotional distress, causal perceptions, qualitative, mental health

## Abstract

**Background:**

Help-seeking is intrinsic to efforts to manage the onset, maintenance, or escalation of mental health difficulties during adolescence. However, our understanding of adolescent help-seeking remains somewhat nebulous. A greater comprehension of help-seeking behavior from the perspective of adolescents is needed. It is also prudent to explore help-seeking behavior in the context of perceived cause for emotional distress, particularly as causal beliefs have been found to influence help-seeking behavior in adults.

**Objectives:**

The present study sought to categorize adolescents' experiences of help-seeking, and to examine the extent to which these categories (or “types”) of help-seeking behavior are associated with their perceptions of causal factors for emotional distress.

**Methods:**

The data for this study were drawn from interviews conducted as part of the HeadStart Learning Programme. The sample comprised of 32 young people aged 11–12 years. Ideal-type analysis, a qualitative form of person-centered analysis, was used to construct a typology of adolescent help-seeking. Participants' help-seeking “type” was then compared with their perceived cause for emotional distress “type.”

**Findings:**

We developed four distinct categories of help-seeking: (1) guided by others who have taken notice; (2) skeptical with unmet needs; (3) motivated and solution focused; and (4) preference for self-regulation. Simultaneously, we identified principal associations between perceived cause of emotional distress—(1) perceived lack of control; (2) unfair treatment; (3) others: their actions and judgements as the catalyst; (4) concern for self and others; and (5) self as cause—and help-seeking approaches. “Perceived lack of control” was most likely to be associated with “others who have taken notice”; “Unfair treatment” with “skeptical with unmet needs”; “others: their actions and judgements as the catalyst” with “motivated and solution focused”; “concern for self and others' with ‘guided by others who have taken notice”; finally, “self as cause” was most likely to be associated with “preference for self-regulation.”

**Conclusions:**

This study demonstrates meaningful and distinct categories of adolescent help-seeking and offers empirical evidence to support the assertion that perceived cause for emotional distress may influence the help-seeking approaches of adolescents.

## Introduction

Help-seeking is intrinsic to efforts to manage the onset, maintenance, or escalation of mental health difficulties during adolescence (1). However, our understanding of adolescent help-seeking remains somewhat nebulous (2). A greater comprehension of help-seeking behavior from the perspective of adolescents themselves is crucial to better support at-risk young people on their terms, and such understanding can support the continued development of prevention research and practice (3). Given the propensity for emotional distress during adolescence (4), it is also prudent to explore help-seeking behavior in the context of perceived cause for emotional distress (5), particularly as causal beliefs have been found to influence help-seeking behaviors in adults (6–9). To address this need, the present study sought to categorize early adolescents' experiences of help-seeking, and to examine the extent to which these categories (or “types”) of help-seeking behavior are associated with their perceptions of causal factors for emotional distress. The categories of perceived cause for emotional distress were established with the same sample in an earlier paper (5) and included: (1) perceived lack of control; (2) unfair treatment; (3) others, their actions and judgements as the catalyst; (4) concerns for self and others; and, (5) self as cause.

Adolescence is defined as the stage between age 10 and 19 (10). It can be split into three distinct phases, early adolescence (10–13 years), middle adolescence (14–16 years), and late adolescence (17 + years). This epoch is a key developmental period, with great potential for self-development as well as physical and intellectual growth (11). It can also be a challenging period given changes across numerous developmental domains (12), which are often emotionally demanding (13). Heightened emotional reactivity is a quintessential marker of this period (14), and emotional regulation, our ability to effectively respond to and manage our emotions, is undergoing its own process of development (15). Research indicates that internal regulatory strategies change throughout the period of adolescence, moving from limited efficacy in early and middle adolescence to a greater reliance on adaptive strategies later in the epoch (15–17). Taken together, it is perhaps unsurprising that we typically see an increase in experiences of emotional distress (i.e., difficult affect responses, such as feeling angry, worried, anxious or depressed), thought to indicate burdensome or ineffective adaptions to environmental demands (4). Most of us have experienced challenging affect responses to some extent, especially during our teenage years (18). For many adolescents, elevated emotional distress will not lead to significant difficulties; however, for some, heightened emotionality may contribute to the onset of life-impacting difficulties (14).

Adolescence is a critical period for the onset of mental health difficulties, with emotional difficulties such as anxiety and depression among the most prevalent (19–21). The number of adolescents aged 11 to 16 in England reporting a “probable mental disorder” increased from 2017 to 2020, prevalence has remained relatively stable at a rate of 20.4 percent since, whereas prevalence among 17 to 19 year olds has risen from 17.4 percent in 2021 to 25.7 percent in 2022 (22). This increase may have been exacerbated by the COVID-19 pandemic (23), but concerns regarding the trajectory of adolescent mental health, across varying levels of difficulty, were present prior to the pandemic (24), on a global scale (20, 25). Correspondingly, there is significant interest in early intervention to curtail the onset of life-impacting difficulties. A concurrent goal is to improve adolescent wellbeing, a construct broadly referring to personal and social functioning, with a focus on feelings and life evaluation (26). Whilst aspects of wellbeing, such as pleasant emotions and meaningful engagement (27), reflect aspects of good mental health, mental health difficulties and wellbeing have distinct correlates (28). Therefore, the absence of disorder does not necessarily indicate wellness and adolescents who are not experiencing clinical levels of difficulties may still need support.

Unlike behavioral problems, emotional difficulties are more easily concealed, making it difficult to identify a young person's internal struggle (29, 30). As a result, articulating the need for help has been identified as an important factor to support adolescent mental health and wellbeing (31). However, at a time of peak vulnerability, adolescents are the least inclined developmental group to seek professional help when it comes to mental health concerns (32). There are numerous factors that influence one's ability to explicitly ask for help for emotional difficulties. Qualitative work has helped us to understand wider factors influencing the help-seeking in greater detail. Recent work by Westberg et al. (33), for example, demonstrates that adolescents may be unfamiliar with or insecure in the processes of help-seeking. Adolescents may also feel that current structures of support are inaccessible and unresponsive (33). Thus, this more nuanced insight helps to dismantle the prevailing deficiency focus on adolescents as “poor” help-seekers (34), and instead highlights that wider contextual factors might be impinging on this process. Indeed, to effectively address mental health challenges among adolescents and young adults, it is crucial to understand how they perceive and experience support and seek help (35, 36).

Help-seeking has the potential to modulate the severity and persistence of problems (37, 38) and can support timely intervention and recovery (39, 40). Despite this importance, there remains disagreement about how we define help-seeking (41). According to Rickwood and Thomas (42), it is “an adaptive coping process that is the attempt to obtain external assistance to deal with a mental health concern” (p. 180). This is split into formal help (e.g., clinicians) and informal help (e.g., teachers, friends, and family). Evidence indicates that adolescents tend to favor more informal support, particularly friends (38, 43). In a similar vein, we know adolescents are more likely to initially share difficulties with their peers, and peers can be extremely valuable to the help-seeking process (44). However, a meta-analysis examining association between informal help-seeking behavior and adolescent psychosocial outcomes would suggest there is a paucity of research investigating links between informal help-seeking and psychosocial outcomes during adolescence (41). Qualitative research shows us that, whether formal or informal support, adolescents want to be heard by adults they seek help from, but often do not have this experience when it comes to seeking support (36). Barker (45) states that help-seeking refers to *any* steps taken by an adolescent, who feels they need assistance, to meet their needs in a positive way. Barker's (45) definition allows for us to consider that adolescents may be going to great efforts to seek and engage with varied forms of help; therefore, it is valuable to explore help-seeking from their perspective as experts in how they manage challenges to their wellbeing (36, 46).

Whilst help-seeking is considered to be inextricably linked to intervention and prevention efforts for mental health difficulties, low levels of help-seeking during adolescence are a significant barrier to this effort (1, 40, 42). The process of seeking help is complex, with multiple decision points as well as barriers and facilitators that can impact progression (32). There is a growing body of research exploring what supports or hinders help-seeking during adolescence [e.g., (37)], with the most identified barriers including stigma and negative views toward services and professionals (39). Midgley et al. (47) suggested that causal beliefs may also have consequences for adolescent help-seeking. Indeed, Aguirre et al. (39) explain that the nature of mental health symptoms, such as self-blame and the experience of emotional distress, may contribute to diminished help-seeking behaviors. Yet, our understanding of the ways that perceived cause for emotional distress might influence help-seeking during this developmental period is limited, particularly in relation to young people considered to be at-risk of mental health difficulties.

## The current study

This study builds on our previous work where we identified categories of perceived cause for emotional distress among adolescents considered to be at-risk of developing mental health difficulties (5). We identified five distinct “types” of perceived cause using ideal-type analysis, a method for developing categories from qualitative data, including: [1] perceived lack of control; [2] unfair treatment; [3] others, their actions and judgements as the catalyst; [4] concerns for self and others; and, [5] self as cause. Our goal here is to identify potential patterns across types of perceived cause, as identified in O'Neill et al. (5), and types of help-seeking, identified in the present paper. Hence, the aim of the present study was 2-fold: (1) to explore and identify meaningful categories of help-seeking behavior among adolescents with personal experience of emotional distress; and (2) to examine the extent to which causal perceptions for emotional difficulties are associated with help-seeking behaviors.

To our knowledge, no other research has attempted to achieve these dual aims within an early adolescent sample. We aim to enhance understanding of the interplay between perceptions of cause and seeking help for emotional distress in early adolescence. Adolescents face a multitude of challenges (12), thus, our aim was to determine potential overarching patterns in their approach to help-seeking rather than focusing on singular instances. Help-seeking is complex (32). In attempting to develop this typology, it was not our intention to negate this complexity, but rather to explore potential commonalities as well as differences in approaches. Knowledge of overarching patterns has implications for how we work with adolescents to better understand their approach to seeking help so that we might facilitate a process that is aligned to their experiences, needs and expectations.

## Method

### Setting for the study

Beginning in 2016, the 6-year HeadStart Learning Programme was implemented with the intention of exploring and testing ways to improve the mental health and wellbeing of 10–16-year-olds. In doing so, the programme aimed to prevent the development of significant mental health difficulties by implementing a plethora of preventive interventions at six sites across England. The HeadStart Learning Team were tasked with assessing the impact of the varied interventions in the context of broader issues related to adolescents' mental health and wellbeing, coping behavior, and experiences of professional and social support. The longitudinal evaluation of HeadStart consists of survey and interview data collection, gathered annually. Interview data from the Learning Team's evaluation of the HeadStart Learning Programme were used in the present study. It is important to remember that this study has been conducted in England, a Western setting, and to bear in mind that help-seeking and explanatory models of mental health and wellbeing may vary depending on the cultural context (48, 49).

### Participants

The sample was drawn from 82 young person interviews, conducted as part of the first annual wave (2017) of qualitative data collection. The evaluation team asked school or HeadStart staff to invite adolescents who *could* receive or who *had* received support (including targeted or universal) from HeadStart to take part in the interviews. As outlined, the present work sought to build on findings from earlier work by the authors; therefore, the present study is framed as a “companion” paper for O'Neill et al. (5), and the 32 interviews used in both studies are the same. The initial sample of 32 was chosen based on discussions of perceived cause for emotional distress and lived experience of emotional distress. Initial decisions around inclusion were made by AON, who logged reflections which were reviewed and discussed with ES who had extensive knowledge of the interviews. Of the initial 82 interviews, 11 participants were excluded due to age (9–10 YO). The remaining transcripts (11–YO) were checked for prevalent discussions of personal emotional distress as well as perceptions of cause for this distress. Those who discussed both were included in the study (*N* = 32). Adolescents who reported situations that might be perceived externally as distressing but did not report feeling distressed themselves were not included. Likewise, those who indicated distress but not a perceived cause were excluded; excluded participants were checked and verified by ES. It is recognized that this approach to participant selection may mean that experiences of help-seeking in the sample more broadly were not included in this study. However, as well as exploring categories of help-seeking behavior, we were interested in understanding the extent to which overarching causal attributions might correspond with global help-seeking behavior. Utilizing the same sample was intrinsic to this goal. The final sample consisted of 32 adolescents. The age range was 11 years and 8 months to 12 years and 9 months [mean (M) = 12 years, standard deviation (SD) = 0.31].

### Ethical considerations

The qualitative strand of the Learning Team's national evaluation of the HeadStart Learning Programme received ethical approval from University College London (ID Number: 7963/002). Adolescents were given the opportunity to participate in the interviews, which they were free to accept or decline. Researchers conducting the interviews had up-to-date Disclosure and Barring Service checks and received safeguarding training from their host institution. Before adolescents who decided to take part were interviewed, they and their parents were asked to read a participant information sheet detailing the study. Thereafter, informed consent was sought by parents/carer(s), and assent to take part was sought from the participants before the interviews began. Interviewees were made aware that the information they provided would remain confidential, unless they disclosed something that indicated harm to themselves or others. During the write-up phase, all identifying information was anonymized; interviewees were aware this would happen.

### Data collection

Data were generated through one-to-one semi-structured interviews with the adolescents. Interviews varied in length from 15 to 60 min (M = 38.02, SD = 9.85) and were conducted by the research team in a private room at the participant's school. An interview schedule was used, which was developed as part of the programme evaluation, in collaboration with the research team and Common Room—A consultancy and advocacy organization supporting the views of adolescents in research and policy. The schedule allowed for a focus on experiences of coping with and receiving support for difficulties, including their experiences of and opinions on support from formal (e.g., from the HeadStart programme or other professionals) and informal (e.g., from family and friends) sources. The broad contextual focus of the interview schedule covered important domains in the lives of the adolescents, including school, home, family, friendships, and feelings/emotions.

The main purpose of the interviews was to support the evaluation of HeadStart rather than to directly explore help-seeking behavior or causal attributions for emotional distress. However, during the interviews, adolescents were asked about their experiences of coping and receiving support when facing difficult situations and feelings in life. Therefore, they often spoke about help-seeking and causal perceptions either due to a prompt from the interviewer for further detail or spontaneously in the context of their narrative. The interview schedule lent itself well to such explorations, and perceptions of cause for emotional distress in the context of help-seeking has relevant implications for adolescent wellbeing and the efforts of HeadStart.

### Data analysis

Our aim for this study was 2-fold: (1) to explore and identify meaningful categories (“types”) of help-seeking behavior among our sample of adolescents with personal experience of emotional distress; (2) to consider the extent to which overarching perceptions of cause for difficulties and type of help-seeking behavior might overlap for the participants in this study. Accordingly, ideal-type analysis was chosen as the most suitable form of analysis. This qualitative approach offers a systematic methodology for developing a typology, the grouping of participants with shared features (50), from qualitative data and allows for comparative analysis between and within clusters of cases (51). Categories or “ideal-types” were derived inductively or bottom-up from the data and participants were placed into the category which best represented their global approach to help-seeking. This approach enabled us to offer distinctions between overarching help-seeking behaviors, which is beneficial given the complex and varied nature of help- seeking. An ideal type essentially describes the bringing together of influential attributes from similar cases to explore how a particular phenomenon is understood (52, 53). Using Stapley et al.'s (54) seven steps for ideal-type data analysis, the following procedures were observed:

Step 1: Becoming Familiarized with the Data Set. The first and second authors (AON and ES) were already acquainted with the data from work conducted earlier (5), conducting the interviews (ES), and reading the transcripts and listening to the audio files from the interviews several times to purposefully familiarize themselves with the data (AON and ES). From the beginning, precautions were taken to minimize bias in interpreting the data, particularly given AON and ES' knowledge of future timepoints and extensive involvement with the analysis for the sister paper. These precautions included involving multiple co-researchers throughout the analysis and ensuring that findings were grounded in the data. As an additional step, we invited new members to the team who were not involved in the companion study, one of whom had no knowledge of the interviews from alternative timepoints.

Step 2: Writing the Case Reconstructions. A case reconstruction, or summary, of each interview was created by AON, focusing on instances related to help-seeking behavior. Each summary was a description of the relevant content from each interview transcript. Summaries were checked and verified by AON against the full interview transcript and reviewed by ES.

Step 3: Constructing the Ideal-Types. Case reconstructions were systematically compared to identify patterns across participants' experiences. This was completed initially by AON and then again with a team comprising of the first author and a co-researcher. This co-researcher had no involvement in the companion paper. It was essential to have someone who had not seen or been involved in the companion paper to minimize unintended bias when exploring links between the findings of the present and companion paper. After initial discussions, AON grouped similar cases together to construct the ideal-types.

Step 4: Identifying the Optimal Case. A case from each cluster was chosen that best illustrated the ideal-type; this case then acted as an orientation point for comparison of other cases within that type. The presence of an optimal case for each cluster allowed us to explore the extent to which participants shared common experiences, whilst also identifying divergence within each group.

Step 5: Forming the Ideal-Type Descriptions. A detailed description of each ideal type was constructed, with the optimal case for each type in mind. The intention was that all cases categorized within each type could be identified using the type description.

Step 6: Credibility Checks. Echoing other work utilizing ideal-type analysis (5, 51, 55, 56), we carried out extensive credibility checks to ensure that the ideal-types and descriptions clearly reflected the data. This stage involved input from three co-authors, including the first author, and an independent researcher. AON's grouping of similar cases together to construct the ideal types was reviewed by the third author (IR), the placements were discussed and refined based on IRs feedback.

To verify our sorting, the independent researcher proceeded to place each case under the most befitting type without prior knowledge of placement. Initial consistency between the placements was <100%. Where there was inconsistency between the placements, the ideal-type descriptions were refined to ensure clarity and cases were recategorized within alternative clusters as necessary. Discrepancies were reviewed by ES and discussed with AON. Final placements were discussed extensively between AON and ES, resulting in the loss of an ideal_type. It was determined that one of the types was not operating effectively as an independent category. Accordingly, the features were collapsed into the appropriate type and the participants recategorized, leaving us with four rather than five groups. The final categorization between AON and ES was done with 100% agreement.

Step 7: Making Comparisons. Once the similarities between cases were clearly delineated, variations within the clusters were explored to illustrate the nuance within the types.

## Findings

Based on the interviews given by participants, we identified four overarching types of help-seeking behavior, including: [1] guided by others who have taken notice, [2] skeptical with unmet needs; [3] motivated and solution focused; and [4] preference for self-regulation. The four types are presented below alongside ideal-type descriptions, the optimal case from each group, and a summary of other cases within the cluster. The participant names provided below are pseudonyms.

### Ideal-type 1: guided by others who have taken notice (*N* = 10)

#### Ideal-type description

The adolescents in this category often highlight the ways in which their approach to help-seeking is evolving due to initial offerings of support, including being referred for intervention support. Typically, adolescents here discuss past experiences of coping less favorably and emphasize that they now deal with things “*better*” owing to support offered. For this group, someone taking notice and offering help gives them a safe space to share, as well as access to a person who they trust to support them; if offered a support structure that provides trust, privacy, and a sense of feeling cared about, they will likely continue to engage with it thereafter. The importance of this is highlighted by an inclination to avoid addressing certain difficulties in their interviews, as well as a broader focus on trust. There is a sense that these adolescents know that they need support, but they may not feel able to reach out or may feel uncomfortable accessing it without guidance. However, they are likely to share their difficulties with people who they are close to who might be able to facilitate the help-seeking process. There is often a focus on having someone who they are close to “*sort things out*” or seek help from others on their behalf (e.g., telling a sibling, friend, or parent about an issue so they can bring it to a teacher on their behalf). They are willing to share and engage with help—perhaps more reactively than pro-actively—but facilitation helps them take the first step, otherwise support is only sought at a breaking point. Essentially, they are in the early stages of figuring out what works for them and need help to do so.

#### Optimal case

Maggie is presented as the optimal case for this ideal-type. Maggie tells us about her experiences of violence at home. She indicates that this has been happening for a long time, and when she finally told the teacher about it, this made “*it easier for [her], to not feel... like [she's] going to have to put up with being hit*.” The catalyst for finally sharing was someone recognizing and reporting that she had been self-harming due to stress. She found not having to keep everything a secret anymore a relief and she felt like finally talking to someone was the reason that the self-harming “*stopped*.” Maggie has spoken to her sibling about being bullied in the past, who helped her to talk to school staff about it, which made her “*feel better”*.” In general, she finds it easy to talk to her sibling about her problems because of the “*the fact that [her sibling] says [they] will try and get it sorted out and [they know] quite a lot of people in the school.”* She also speaks to her mum about things that she finds difficult; her mum helps her by calming her down and sorting things out: “*If it's something about school that's upset me she will phone the school to get it sorted out and if it's something at home she will tell the person to stop and leave me alone*.”

#### Summary of the other cases within this type

Before having a peer mentor, there were difficulties that Ian had not spoken about; he has found having a space to do this helpful, especially as his mentor is “*a very nice person*” who is easy to talk to. Avery and Thomas had similar experiences. Jayden has had support made available to her after speaking with a teacher, as a result, she is speaking with someone about her difficulties at home and is going to have further support. Like other adolescents in this group, Joseph and Riley have valued having a safe space made available to them to facilitate sharing their experiences and concerns. They have been participating in the same type of small-group psychoeducational sessions, and Joseph feels “*it's a great place, […] and it makes you open up and it makes you know the emotions*.” Riley also feels that participation in this activity is where she “*learned to talk to somebody*” about her difficulties. Similarly, Quinn attends a youth club that gives him a space to share, which acts as a steppingstone to support: “i*f you've got any problems, you can speak to them*” and they can “*sort it out”;* he will only share with people he trusts. Frankie has had a similar experience, but for him he “*might be able to talk about once a year, just in case something big happens and [he needs] to talk to someone*.” He feels that it is helpful doing this because then he knows that the problem is going to get “*sorted out at one point*.” He alludes to problems he is facing, but when asked if he would like to speak about them, he responds, “*I'd rather not at the moment*.” Parker also values having someone to sort out problems; sometimes he wants to leave school because he has “*loads*” of “*upset inside*,” instead he finds a teacher to speak to which takes the “*emotions away*.” Parker had intervention support organized for him because he was “*getting quite angry and reactive*” and feels this support has helped him “*know what to do*” when he is facing difficulties.

### Ideal-type 2: skeptical with unmet needs (*N* = 5)

#### Ideal-type description

Adolescents in this category often recall times when they did not feel supported in the ways that they wanted or needed when they actively sought help. This appears to have led to a sense of skepticism and even apathy in relation to seeking help. Often adolescents in this group will talk about issues they are having and how they feel like they just “*have to get on with it*.” This is different to not ruminating on the problem and trying to move forward, but rather a ‘what is the point' mentality. Some may even turn down support offered to them because of bad experiences in the past or no longer share their problems due to a previous experience of trust being broken. Young people here may indicate that they can get support but not when they really need it, this might be because they feel that other people will be busy at the time of greatest need. There is also a sense of not feeling heard or fully understood when they have tried to reach out for help, or a fear that they would not be taken seriously if they did. The adolescents in this group tend to appreciate friendships that offer them a distraction from the problems that they might be facing, and there may be a reliance on others to make them feel happy. This gives a sense of wanting to forget about or be distracted from the problem that they are facing, a sort of escapism from things that they may feel cannot or will not be resolved.

#### Optimal case

Arden represents the ideal-type for this group. Arden explains that she is being bullied and when asked how she copes, she responds: **“***Just go along with it, because I'm used to it*.” She recalls a time her teacher tried to intervene with classmates calling her names because she was “*crying every day*.” This backfired because it inadvertently led to further bullying. Arden feels that teachers have not always supported her effectively, this includes taking other people's sides over hers: “*Favoritism, I think it is. I don't know why*.” She spoke about a specific incident of being bullied by a peer, during which she was not believed when she told the teacher, leaving her to manage it herself: “*I just had to deal with it, because no one did anything*.” She talks about a few teachers and friends who she feels have been supportive, but also states that she cannot trust anyone, “*[c]an't trust no one. I don't trust anyone*.” Arden mentions that there are two teachers who she can go to for support, but she feels that this is not enough and is not sure how to access additional support: “*I do need, like, more support […] Because, like, there's like… I don't feel there's nowhere I can go to. Like, I'm lost for who to go to*.” Despite this perception of having nowhere to turn, when asked if she would like the school to be informed of her difficulties, she responds: “*I don't know*” and explains that things have gotten somewhat better anyway.

#### Summary of the other cases within this type

Kit has spoken to teachers about peers being rude and inappropriate to him at school, “*but sometimes they won't help*.” He no longer feels okay about talking to teachers, instead he feels he has to let his peers carry on with the behavior and try to ignore it. Similarly, when Alex is not feeling happy, he would try to “*deal with it*” and “*try to get over it*,” he feels that going to a teacher to ask for help might make things worse. He also feels that when something “*really desperately needs handling […] there's no one there*” and indicates that teachers are too busy elsewhere to help when they are needed. Carey feels that some teachers do not listen to her, she does not know why this is. She talks about feeling depressed quite often and how no one understands her when she is feeling this way, including herself. She thinks talking to someone when she is feeling depressed might help, but she does not do this. She will avoid talking to her mum about feeling depressed because she has “*really different feeling than [her mum] does*.” For Sam, his friends make him feel better when he is feeling angry, sad or anxious by joking around and distracting from what is going on. When asked if he would recommend people speaking to someone if they are experiencing difficult emotions he states: “*no ‘cause they wouldn't listen*.” He has “*family problems*” and when asked if he has had any support in relation to this, he states “*don't see it as something that can be helped*.”

### Ideal-type 3: motivated and solution focused (*N* = 8)

#### Ideal-type description

The adolescents in this group appear to be motivated help-seekers who are looking for solutions to their experienced difficulties in a way that feels most useful for them. This group appears to be the most invested in the process of *active* help seeking, often seeing asking for help as a strength. Therefore, even if people let them down, they are not dissuaded, but rather motivated to look elsewhere. Like the guidance group, they perceive previous behaviors in the context of their difficulties as problematic but tend to feel like they have developed better ways of coping; however, they are almost a step further along than the guidance group in that they are more able to *proactively* help seek. Here, the adolescents tend to be clear on their preferences for help-seeking, e.g., a preference to share with people their own age due to perceived shared understanding, or a preference to express themselves creatively to process and deal with difficult emotions. They may face barriers in relation to help-seeking, such as not being believed or taken seriously, but, unlike type two, they are more likely to persist until they find a solution. They value good quality support and will talk about this with fondness and gratitude, and they tend to focus more on the ‘good support' rather than on times when their needs were not met.

#### Optimal case

Kris represents the optimal case for this ideal-type. Kris describes a close relationship with friends that enables her to share her difficulties; she feels that even if she only told them “*a little bit*” they would “*understand really well*.” She finds her friends “*easiest*” to talk to, therefore she does not like falling out with her friends and losing this kind of support. She mentions having a peer mentor and several teachers at school who she feels like she can also talk to if she needs help. Of her peer mentor she states: “*I feel like we're gonna be able to talk to each other a lot more ‘cause we're both young*.” Kris feels that she can utilize the support of her peer mentor so she does not have to worry about “*friends not being there*” because she can “*always go*” to her peer mentor instead. In her opinion, it is harder to talk to older people about problems because you have “*explain it to them a lot more*,” but when talking with friends you can be a “*bit less formal*.” There are also teachers she will talk to in school if needed, she explains that one of the teachers is “r*eally nice*” which is why she feels she can talk to her.

She also talks to her mum about her difficulties and prefers to speak to her rather than her dad because she feels her mum understands a lot more. However, under certain circumstances she will speak to her dad, but it has to be “*for a really good reason*” that will help her “*get something off [her] chest*.” When she argues a lot with one parent, she runs to her other parent's house who she feels is the only one who understands. Kris also explains that she has experienced abuse from a family member. She spoke to someone in a weekly group support session about this: “*I told the lady (Person B) about it and, it just felt like I could get it off my chest, so that's somebody actually new*.”

She also received support from mental health professionals outside of school, which she “*really enjoyed*”; in explaining why she enjoyed it, she states: “*Cause once I got to know the person who I was talking to […] it felt like I was just in my own home and I could just like, I don't know it just felt like I didn't have to like sit there and talk to her directly so I could like, go and play while she was talking to me and it felt like I could just relax*.” She explains that she might be going back to this support because she felt like she could talk to them a lot easier and because she feels she needs a bit more support. She believes it will help her “*mentally*” and she will not have to “*bottle it up or anything*.”

#### Summary of the other cases within this type

Like Kris, Annie has supportive friends, and she feels that they have a “*strong bond*.” She likes to draw to express her emotions and feels like music helps her through difficult times, making her feel “*not alone*.” Though she would talk to her dad, she prefers to speak to her mum as she feels that she can “*relate to most things*,” and she believes that talking to people about difficulties is helpful. Shae also feels like her friends are always there for her, in fact she is able to list a number of people who she would talk to if she was feeling upset, including her parents, HeadStart intervention staff and her siblings. Luca talks extensively about worries in relation to various aspects of her life and she is very focused on resolving her worries. She engages with various systems of support to do so, and she makes a great effort to enact the advice that she gets from her family and support worker. Archie also talks to her teachers and family about her problems, she feels like they give her “*good*” advice, which she does her best to enact. Jodie discusses support across different areas of his life, and he actively engages with this support. He works hard to overcome barriers when seeking support, including when school staff are not as supportive as they could be. Like Jodie, Jamie finds ways to overcome challenges when it comes to seeking help, this includes writing in a journal for her mum to read so that it does not distract from support she feels like her sister needs. For Blake, her parents and friends are a trustworthy and strong source of support, and she is also comfortable talking to teachers about her difficulties: “*[b]ecause, um, that's [the teacher's] job basically, in the school, 'cause they wouldn't hire someone, who, erm, isn't good at that*.”

### Ideal-type 4: preference for self-regulation (*N* = 9)

#### Ideal-type description

This group of adolescents appear to favor self-regulation to manage their difficulties. There is not necessarily an aversion to other forms of seeking help, but rather they feel capable of or have a preference toward managing themselves and their reactions. They may take responsibility for their feelings and responses, determining that they are old enough to deal with problems themselves. Adolescents here may talk about having control over their feelings, or desire to control feeling supported by an aversion to ruminating on problems. There is a possibility that they might use tactics to self-regulate in order to mask how they are really feeling to avoid impacting others; for instance, to protect others from the consequences of their anger or to protect their family from their true feelings. They typically have a good sense of where they could go for help but tend not to explore these options. However, they appear to be aware that there might be times when they need to talk to others if it is deemed serious enough. Should they reach that point, there is a focus on finding the most appropriate person for the situation (e.g., issue in school requires a teacher, issue at home requires parent). For adolescents in this group, there is a sense of wanting to be alone, to calm down, and return to “*normal*” when emotions are heightened. This process might involve things like coloring, listening to music, as well as yelling and punching. Adolescents in this group have tried various methods recommended by others, but they tend to rely on themselves most frequently and typically find this to be the most effective or preferred solution.

#### Optimal case

For this ideal-type, Craig is the optimal case. To manage his anger, Craig lists numerous strategies: “*Like, I start moaning. Like I don't do it that much in school, but if I'm at home I might do it. Um, I'd shout or something like that, like walk outside, and I like playing football, so I like kicking the ball straight at, like, the bricks and the thing, like, of the house just to make as much noise as I can*.” He says that this does not help and annoys his family. Craig feels that teachers would support him to the best of their ability, but he does not feel like he is upset often, he believes he can control how he feels and thus “*always feel happy*.” When asked how he controls feelings, he responds: “*I just don't think about it […] you could like have something said to you that upsets you but you just don't think about it a lot*.” When he is upset, he does not talk to anyone, and instead will “*sit there*” in his room, and play video games “*like a normal person*.” He typically does not talk to anyone because he feels like it is usually something that he can deal with himself: “*Cause like it could be for stupid reasons and, like, I can deal with it myself, like I'm old enough to deal with it myself instead of like, bringing other people into it*.” In his view, he should always deal with his problems himself, however, if he were dealing with a significant problem, then he might want to speak with someone “*sometimes,”* but this is only under certain circumstances: “*if it's serious, I might but, like, sometimes I'd be able to control it. If it's serious, serious. I'll only tell certain people, like someone I've known all my life, I'd only tell them*.”

#### Summary of the other cases within this type

Darragh feels that he is “*very implosive*” and so he does not really “*show a lot*.” He indicates that he likes to hide emotions, particularly anger: “*I can keep it in me but like but I'm not sure when it is going to burst*.” He thinks about good times that he has had with a person or manipulates his body so that he cannot punch someone and to keep the anger “*in*.” Dale also deals with his worries by thinking about all the good times that he has had with his friends. When he feels sad, he will “*just wait until it's all gone out and [he] can start feeling happy again*.” He explains that the length of time this takes depends on “*how big the matter is*.” He has a number of activities to help him through difficulties, but this does not help when the problem is “*really big*” because he does not “*really want to be happy in a time of sadness*.” When asked if he would speak to someone when feeling this way, he explains, “*I'd normally just keep to myself unless it's not that big, but normally it is*.”

When she is upset, Emilia wants to be alone, “*because then [she] can like calm down easier if [she's] angry, [and] just go back to normal*.” She has self-regulating strategies in place to help this calming process, including watching YouTube videos, coloring and texting friends. When asked if she would like any support for feeling upset or angry, she responds “*Not really, I'll be fine*.” Bobbie also feels that doing things on your own can help you calm down, he indicates his friends, bike and dog help him do this. Aoife, on the other hand, punches when she is angry, “*I punch my pillow and throw things at my wall*,” but she also lists a multitude of activities she engages in when she is having a hard time, including music and drawing which help her to regulate. When asked if there was someone who she would talk to when having difficulties, she responds “*no because I sort it out myself* ,” but she will speak to someone if it is “*really, really important*,” though not strangers, as they are “*off limits*.”

Lewis likes to chat with friends and do “*bloke things*” with them. He uses “*breathing out the mouth and in through the nose*” and a stress ball to calm down. When he needs to calm down, he will walk away from the person causing the issue, he feels like calming down is what works best for him. For Liz, reading extensively helps her feel better when she is stressed. Of reading she states the following: “*I like forget what's going on in my life and feel a lot better*” and explains that the stress goes away when she reads. To manage his difficulties, Finley tries to “*channel it out […] just like trying to like think of different stuff and forget about it.”* He talks about experiences of anger and how he manages this: “*I guess breathing is a good one; just kind of breathing out*,” and reiterates his preference to focus on being happy. He feels like talking about family problems is difficult, owing to familial loyalty and the “*bond*” that you have with parents.

### Categories of help-seeking and corresponding perceived cause of emotional distress

Participants' help-seeking types, identified in the present study, and corresponding category of perceived cause for emotional distress (perceived lack of control; unfair treatment; others: their actions and judgements as the catalyst; concern for self and others; and, self as cause), identified in our earlier study (5), are outlined below. Table 1 illustrates the number of participants in corresponding types. This information, and the accompanying commentary presented below, are only intended to be exploratory as we note that the nature of the study and sample size precludes more formal inferential quantitative analysis.

**Table 1:** Participant corresponding categories of help-seeking and perceived cause.

**Categories of perceived cause for emotional distress**					
	**Perceived lack of control**	**Unfair treatment**	**Others: their actions and judgements as the catalyst**	**Concern for self and others**	**Self as cause**
**Categories of help-seeking**					
Guided by others who have taken noticed	3	0	3	2	2
Sceptical with Unmet needs	1	3	1	0	0
Motivated and solution focused	0	1	5	1	1
Preference for self-regulation	0	2	3	1	3

## Discussion

This study sought to capture overarching approaches to help-seeking among adolescents considered to be at-risk of experiencing mental health difficulties. Concomitantly, we were interested in exploring the potential overlap between categories of help-seeking and perceived cause of emotional distress. Using interview data from adolescents with personal experience of emotional distress, we developed four distinct categories of help-seeking: (1) guided by others who have taken notice, (2) skeptical with unmet needs; (3) motivated and solution focused; and (4) preference for self-regulation. We also compared the help-seeking type that our participants exhibited to their perceived cause for emotional distress type—(1) perceived lack of control; (2) unfair treatment; (3) others: their actions and judgements as the catalyst; (4) concern for self and others; and, (5) self as cause, as identified in O'Neill et al. (5), and reported the patterns observed. Whilst there is variability in the corresponding percentages across the ideal types (see Table 1), here we report the principal patterns. We found that participants identifying perceived lack of control as their causal type tended to also identify ideal-type 1: guided by others who have taken notice as their help-seeking type; those in the unfair treatment type tended to align with ideal-type 2: skeptical with unmet needs; others: their actions and judgements as the catalyst with ideal-type 3: motivated and solution focused; concern for self and others with ideal-type 1: guided by others who have taken notice; finally, those identifying self as cause tended to also align with ideal-type 4: preference for self-regulation. These findings suggest that overarching causal perceptions for emotional distress may have an important bearing on help-seeking, or vice versa; thus, highlighting the importance of taking causal perceptions into account to support help-seeking in the context of emotional distress. Simultaneously, our findings highlight the value of exploring help-seeking from the perspective of adolescents to better understand the nuances of their approach.

### The help-seeking ideal-types and the important role of others

Our findings could support the existence of a continuum from more active to passive help-seeking tendencies or attitudes. For instance, at one end of the continuum are adolescents who are “motivated and solution-focused” and “guided by others who have taken notice,” who tend to actively seek help from others or who share their difficulties with people who they are close to who might be able to facilitate the help-seeking process. Whereas, at the other end of the continuum are adolescents who are “skeptical with unmet needs” and those who have a “preference for self-regulation,” who have a sense of skepticism and even apathy in relation to seeking help or who prefer to rely on themselves to manage their difficulties. The categories that we uncovered further highlight the important influence that others play in relation to where adolescents are situated on this continuum, as well as how they might move within it. From what we have observed, it is possible that there is a point at which the actions and reactions of those who are able to support adolescents might impact the direction and development of their overarching approach to help-seeking. The categories identified, therefore, may be subject to change over time based on previous experience of help-seeking and/or waiting to be noticed. Further research is needed to identify the point at which change might happen and what factors could influence potential change. Overall, the nature of the approaches identified suggest that having someone to check in, take concerns seriously, offer resources, and to co-create a plan of action with adolescents may benefit the help-seeking process.

Ideal-type 1: “guided by others who have taken notice” in particular emphasizes the critical role that significant others have in promoting and sustaining engagement with help-seeking. Here, the adolescents demonstrate an awareness that they could benefit from additional support but do not necessarily feel able to reach out without a prompt to do so, hence there is heightened reliance on others. There are numerous reasons why adolescents may find it difficult to ask for help initially, including stigma, embarrassment (2) and not knowing where to go for help or how to access it (57). These aspects reflect components of mental health literacy, which broadly refers to knowledge and beliefs surrounding mental health difficulties that aid prevention and management (58). While interventions to promote mental health literacy are becoming more available for older adolescents, there remains a comparable lack of interventions for younger adolescents (59, 60). Therefore, initial support may be more prudent during early adolescence where knowledge, skills and an ability to regulate emotions (15) may be less well developed. The participants in this category indicate that they may need additional help in bolstering their mental health literacy, particularly as we observed that having a steppingstone to support can often lead to continued engagement with help-seeking. This kind of support may also be beneficial to the identification of emerging difficulties.

There is prevailing consensus that schools are significantly well placed to support the early identification of mental health difficulties among at-risk students (29). Whilst teachers, parents and caregivers are often instrumental in linking adolescents to clinical care, they are also a valuable support system in their own right (40). Emotional challenges can be difficult to identify (29), and asking for help may facilitate the process of early identification of difficulties and subsequent intervention (31). However, some participants in our study experienced a lack of support when they tried to seek help. For those in ideal-type 2: “skeptical with unmet needs,” external invalidations when trying to seek support often resulted in ambivalence toward help-seeking. This was often due to not feeling believed or taken seriously. Emotional invalidation (e.g., through minimization of emotional distress) in the developing years can have consequences for emotional expression and can play a role in lifelong difficulties (61), our findings suggest that it can also impact adolescents' approach to help-seeking. Therefore, we echo Hellström and Beckman (36) in highlighting that present adults are important to the help-seeking process and that adolescents need to feel heard by adults to facilitate this process.

Whilst previous negative experience of help-seeking has been recognized as a barrier to asking for support (44), we identified variation in the extent to which this might be the case. Participants across types 2 and 3 described past unhelpful experiences when seeking support, often leading to disengagement in ideal-type 2: “skeptical with unmet needs.” However, those from ideal-type 3 “motivated and solution focused” remained enthusiastic about help-seeking despite being let down in the past. It is unclear why this is the case, but a possible explanation may relate to early experiences of support; for instance, effective support early on acting as a buffer against future ineffective experiences. Whilst this is speculation and ultimately beyond the scope of the present study, we do see that in ideal type 1: ‘guided by others who have taken notice' that when effective support is provided, adolescents tend to stay engaged with the process. Another possible reason for the difference may be related to perceived cause for difficulties, which is explored later. What is clear is that ideal-type 3: “motivated and solution focused” demonstrates how adolescents can be active agents in their own recovery and management if appropriately supported to do so. Shared decision making has been promoted across health-care settings (62), it is less well explored in school settings with adolescents considered to be at-risk of mental health difficulties (63). Indeed, it is argued that there is often a deficit focus in relation to adolescence, rather than an acknowledgment of their skills and the enormous potential of this developmental period (64).

The final category, ideal-type 4: ‘preference for self-regulation', includes adolescents who favor using strategies to self-regulate when facing difficulties. Self-regulation, which refers to any regulation by oneself for oneself to facilitate an arrival to a desired state (65), has been linked to adolescents' psychological wellbeing (66). Our findings suggest that some adolescents who prefer self-regulation demonstrate a level of mental health literacy that reflects an awareness of where they can go for help. However, it is likely that they will only access support if they feel that the circumstances are “serious” enough and will often opt for handling problems themselves. Approaches to self-regulation can be sophisticated and less sophisticated; less sophisticated methods might include bottling up emotions (35), this is something some of the young people in this group appeared to utilize. It is important to note that self-regulatory approaches can be taught (35), perhaps suggesting that some young people in this group have had more guidance for self-regulation than others.

### Principal patterns of help-seeking and perceived cause for emotional distress

Midgley et al. (47) argued that aetiological beliefs may have consequences for help-seeking amongst adolescents diagnosed with depression. We echoed these arguments and suggested that perceived cause for emotional distress may influence help-seeking among adolescents considered to be at risk of mental health difficulties (5). The present study sought to empirically explore this suggestion and our exploratory analysis revealed some apparent associations between help-seeking and causal attribution ideal types (though we recognize the need for further work to confirm these associations, which go beyond what was possible in the current study). Further, in drawing these studies together, it is clear that people in the lives of adolescents not only influence causal attributions and help-seeking respectively, but that they also influence how these concepts interact and overlap; perhaps the starkest example of this is that adolescents who identified unfair treatment as their overarching cause for emotional distress were most likely to identify skeptical with unmet needs as their approach to help-seeking. Taken together, this highlights that perceived cause may have the potential to influence approaches to help-seeking, potentially through the role of others and the quality of the infrastructure that they provide for support.

In exploring further the role of perceived cause for emotional distress in relation to help-seeking among adolescents, this work broadly supports our earlier systematic review of the literature (63) where four themes related to causal attribution and help seeking were identified, including: (1) cause and implications for self-preservation; (2) the degree of personal and wider knowledge and understanding of cause; (3) perceived extent of control in managing cause; and (4) cause having potential to affect others. However, findings from O'Neill et al. (63) were drawn from literature that did not explicitly focus on causal attributions for emotional distress and subsequent approach to help-seeking. To narrow this gap, the present study offered this explicit focus by drawing comparisons with our companion study (5). This presented the opportunity to observe patterns and offer a more detailed insight to the link between causal attributions and subsequent help-seeking behavior in the context of emotional distress.

Participants for whom lack of control was the overarching cause of emotional distress were more likely to indicate a help-seeking approach that was guided by others who had taken notice, reflecting a similar lack of control over their own help-seeking behavior. This association indicates an external locus of control, the degree to which one believes circumstances and outcomes are caused by internal or external factors, in relation to both cause and help-seeking. Indeed, perceived extent of control in managing cause was also found as a theme that related to causal perceptions in O'Neill et al. (63). It is, therefore, likely that locus of control impacts causal attributions which may in turn impact their approach to help-seeking. This is further supported by the finding that in terms of the participants who felt that they were to blame for their experience of emotional distress (self as cause), they were also more likely to indicate a preference for self-regulation, suggesting an internal causal locus and a subsequent internal strategy to deal with their problems.

Adolescents identifying others: their actions and judgements as the catalyst as their overarching cause of emotional distress were most likely to be found in ideal-type 3: motivated and solution focused. Although adolescents in this group can face setbacks, for instance not being believed when they share their difficulties, they are focused on finding the best solution for themselves. It is unclear why this might be the case given a potential propensity to find the actions and judgements of others to lead to emotional distress. It is like they are able to see help-seeking as separate from those they are seeking it from and are adept at understanding what works best for them.

Those who identified unfair treatment as the overarching cause of emotional distress in O'Neill et al. (5), were most likely to be categorized as ideal-type 2: “skeptical with unmet needs,” or ideal-type 4: “preference for self-regulation.” This indicates that adolescents who are affected by perceived unfair treatment may carry this through to their help-seeking, which may manifest as either skepticism about how others can help, or explicitly pursuing internalized strategies that do not rely on others. This suggests parallels in terms of dissatisfaction and mirrors previous findings that indicate that an adolescent's ability or desire to communicate their emotions and ask for help can be compromised by a lack of trust (29), and previous negative experiences of sharing emotions with significant others (44). The adolescents in this group often spoke about times when they tried to get help but that they were let down in some way. There is a possibility that the reactions that adolescents in this category received when seeking help or hoping to be noticed were influenced by the receiver's causal beliefs (e.g., people assuming that they are being dramatic). It has been argued that causal beliefs regarding difficulties influence recommendations for help-seeking (67, 68); for instance, research suggests that believing a cause is psychosocial is associated with recommending psychological care (68). There is a possibility that this relates to whether or not people take adolescents' concerns seriously, which we have seen can impact their ongoing approach to help-seeking and feed into their perceived cause for emotional distress.

As for the concern for self and others causal group (5), they were most likely to be found in ideal-type 1: “guided by others who have taken notice,” suggesting that being prone to worrying about impacting others and oneself may cause delays in help-seeking. Numerous studies have observed that adolescents delay or avoid speaking to others about challenges that they are facing, or things that they are finding difficult, to avoid the potential ramifications (69, 70). There may also be a desire not to burden others with their difficulties (71). In our systematic literature review (63), we too found that the degree to which the perceived cause had implications for self-preservation or impacting others influenced help-seeking, although cause having the potential to impact others was only found as a theme for the at-risk group in our review and not the clinical group (63). The present study empirically demonstrates this association once again within an at-risk group.

## Implications

Our findings highlight a number of possible ways to better understand and facilitate the help-seeking process for adolescents at increased risk of experiencing mental health difficulties. First, they highlight the importance of creating space for passive as well as active help-seekers. There is often much responsibility placed on adolescents to seek help rather than a reflection on how this process can be better facilitated. For instance, while it is important to make young people aware of where they can go to seek help and what that help might look like (72), it is also important to have systems in place to help those not actively (or overtly) help-seeking. There is often a deficit focus on not talking, this can be seen as a failure to communicate need rather than a decision that needs to be respected; young people may communicate their difficulties in more subtle ways depending on their perceived cause, and communication is more likely to happen in safe, confidential settings where autonomy is respected (34). Therefore, there is a need to adjust potential deficit perspectives in relation to passive help-seeking. This may help to create a more holistic approach which allows adolescents to access support without them having to ask first. Based on our observations, it is likely that if the right infrastructure is in place, an initial stepping stone into help-seeking will promote continued engagement with the process. It has been noted that offering services within schools reduces the effort required to access other services and may help address structural barriers, including transport and lack of time, for accessing support (73). However, those not experiencing significant difficulties also need places to go and it is important that support is made available across a spectrum of difficulties.

In all considerations related to accessing support, adolescents' views should be seen as intrinsically valuable (35). Therefore, asking adolescents what they would like support infrastructure to look like and ensuring that they maintain agency within this process may be beneficial. To facilitate sharing difficulties, adolescents need to know that their problems will be taken seriously (69), and a lack of understanding on the part of the receiver can lead to dismissive responses (57). Therefore, it is not only valuable to equip young people with knowledge and skills in relation to help-seeking and how to help themselves (73), but also essential to ensure that those most likely to be engaging with adolescents are equipped to offer effective support. This involves enhancing their understanding of the help-seeking process, particularly in relation to causal perceptions. They also need to be aware that self-regulation can be taught (35) and should not just be expected. Likewise, parents, caregivers, teachers and allied professionals need to be supported to validate distress. This might involve being made aware of the extent to which personal beliefs around cause for difficulties might influence their own response and level of support.

Finally, the use of ideal-type analysis to create a typology of adolescent help-seeking behavior is a novel approach, it was also novel with regards to adolescent perceived cause for emotional distress. In using ideal-type analysis in this way, we were able to observe and report principal associations between perceived cause of emotional distress and overarching help-seeking behavior. Whilst more work is needed to explore these associations in greater depth, our findings offer empirical evidence of the existence of an association between perceived cause and help-seeking among adolescents at increased risk of mental health difficulties. This approach allowed us to build on evidence from a previous paper and to synthesize the findings. Understanding the extent to which perceived cause for emotional distress impacts help-seeking has important implications for those working with young people, including understanding their role in the processes of cause and support.

The creation of typologies is a long-established approach to understanding human behavior, particularly to understand and explore similarities and differences in an organized and structured way (50). They can help to identify patterns which ultimately have a bearing on our knowledge and understanding of the world (50). There are a number of influential typologies that have been successful in categorizing complex and varied phenomena. For instance, in providing an empirical basis for attachment theory (74, 75), four categories of attachment have been established, including secure, insecure avoidant, insecure ambivalent (76), and disorganized attachment (77). These categories offer a structured pattern to understand vast experiences of early-life experience and how they might impact later life outcomes. Thus, the categories that have been systematically established in the present research through ideal-type analysis can be used to further our understanding of causal perceptions and help-seeking. Specifically, it has been established that to effectively support help-seeking, we need to consider causal attributions.

### Strengths, limitations, and suggestions for future research

This study contributes original and valuable insight into adolescent approaches to help-seeking as well as the overlap between help-seeking and perceived cause for emotional distress. We offered an exploration in relation to causal beliefs and help-seeking during adolescence, work typically done with adult samples. We also focused on young people at-risk of mental health difficulties rather than those already experiencing serious mental health difficulties, who are typically the focus in help-seeking literature. Young people who are not affected by serious mental health problems still need to have their experiences and views considered; such information is useful when trying to prevent future difficulties and support young people through current challenges (35). This work considered one timepoint in early adolescence, but future research should seek to explore how adolescents' approach to help-seeking changes over time in the context of perceived cause for emotional distress.

We examined help-seeking and perceptions of cause in the broader context of the lives of adolescents, but the interviews used in this study were not explicitly designed for this purpose, meaning that we had less control over probing. Primary data generation explicitly focusing on causal perceptions and help-seeking may be useful (36). The interviews were held with young people who were selected by staff and who agreed to participate in the interviews within a school setting. School and HeadStart staff offered young people the opportunity to take part in the study via expression of interest forms or simple nomination. Therefore, the views of young people who had not been noticed or selected by staff may have been missed in this typology. There may also be other types of help-seeking found in alternative setting with at-risk groups. It is important to note that this study was conducted in a Western context and that there may be cultural variation in relation to help-seeking (49) and explanatory models of mental health and wellbeing (48).

This study was strengthened by the use of ideal-type analysis, which offered a systematic approach to developing the types. This was a novel and useful way to explore the overlap between categories of cause and categories of help-seeking. While we provided a numerical breakdown of patterns observed, we cannot comment on the statistical significance of the associations. However, it was not our intention to validate the types quantitatively. At this stage we were interested in overlap between cause and help-seeking, but it is also important to consider categories that do not overlap and what this might tell us about how and why causal perceptions and help seeking are connected. Exploring associations and lack thereof in more depth is a prudent endeavor for future work in the area.

The types were constructed by the first author, who has been involved in other projects that focus on help-seeking and perceived cause for emotional distress. Their knowledge of the prior studies and broader understanding of the literature has inevitably influenced data interpretation. While there was agreement in the construction of the types among the wider team, and steps were taken to ensure quality and rigor, it is inevitable that wider knowledge will have influenced the construction of the types. This important context within which the types are situated should be noted when interpreting the findings.

## Conclusion

This study demonstrates meaningful and distinct categories of adolescent help-seeking, outlining commonalities as well as variations both within and between the approaches observed. In exploring help-seeking from adolescents' perspectives, this research identified active as well as passive approaches to help-seeking; the knowledge of which is useful for informing intervention efforts as it emphasizes a need to put mechanisms in place to support passive as well as active help-seekers. Simultaneously, we identified principal associations between perceived cause of emotional distress and help-seeking approaches, offering empirical evidence to support the assertion that perceived cause for emotional distress influences the help-seeking approaches of the young people in our study. The understanding that causal perceptions play a role in help-seeking in this context foregrounds the importance of exploring causal beliefs to aid efforts to bolster help-seeking. The combined findings of this research make clear that people in the lives of adolescents not only influence causal attributions and help-seeking respectively, but that they also influence how these concepts interact and overlap. Therefore, the findings underscore the importance of adequate training to help adults in the lives of adolescents understand how their reactions and interactions may inadvertently impact adolescents. Finally, by illustrating a breadth of help-seeking approaches, this work demonstrates the inherent value of considering adolescents' experiences and co-producing solutions with them to enhance the effectiveness of current and future support.

## Data availability statement

The datasets presented in this article are not readily available because access to data is restricted to the HeadStart Learning Team to comply with the study's research ethics approval. The interviews were generated as part of a national evaluation of an early intervention programme and are held by the Anna Freud Center. Requests to access the datasets should be directed to emily.stapley@annafreud.org.

## Ethics statement

The studies involving humans were approved by University College London (ID Number: 7963/002). The studies were conducted in accordance with the local legislation and institutional requirements. Written informed consent for participation in this study was provided by the participants' legal guardians/next of kin.

## Author contributions

AO'N, ES, and NH contributed to conception and design of the study. AO'N summarized the interviews and wrote the first and subsequent drafts of the manuscript. AO'N preformed the analysis, supported by IR and ES to create and refine the ideal-types. All authors contributed to manuscript revision, read, and approved the submitted version.
